# Scoping Review of the Definitions Used to Describe and Understand Harmful Sexual Behaviors in Children and Young People

**DOI:** 10.1177/15248380231218294

**Published:** 2023-12-28

**Authors:** Gabrielle R. Hunt, Daryl J. Higgins, Megan L. Willis, Lottie Harris

**Affiliations:** 1 Australian Catholic University, Banyo, QLD, Australia

**Keywords:** sexual abuse, sexual assault, prevention of child abuse, offenders, youth violence

## Abstract

There is a growing body of evidence that adolescents, and other children, are responsible for a significant proportion of sexual abuse against children. However, there are substantial differences in how this phenomenon is defined and conceptualized between and within sectors. This scoping review explored the current definitions of harmful sexual behaviors (HSB), and other similar terms, used across a range of stakeholder groups. In all, 141 papers were reviewed from both empirical and gray literature sources, including key policy and practice documents. Included papers needed to list a clear definition for the behavior of interest. There was disagreement and inconsistency across the included papers in their conceptualization of harmful, abusive, or problematic sexual behavior (PSB) in children and adolescents. Although the term HSB has been adopted as an umbrella term or continuum in many policy, practice, and research settings, there is a large variance in behaviors, treatment needs, etiology, and harms present across different types of sexual behavior. Relying solely on one term to describe a wide range of sexual behaviors in children and young people may limit the understanding of this issue and imply similarities between groups that are not present. We suggest that clearly defined subsets of HSB, such as sexual abuse, technology-assisted HSB, and PSB, may give more context to the behavior of concern and may be helpful in informing further research, prevention, and best practice approaches.

Recent findings from the nationally representative Australian Child Maltreatment Study revealed that more than one in four Australians have experienced child sexual abuse (CSA) (Mathews et al., 2023). To date, much of the research, policy, and service provision has focused on adults perpetrating sexual harm toward children; however, multiple studies have shown that a significant proportion of sexual abuse has been inflicted by other children and young people in a variety of settings (Gewirtz-Meydan & Finkelhor, 2020). Nevertheless, there is general disagreement and inconsistency about the nature and conceptual framework of this problem. Young people who sexually harmed others previously seemed similar to their adult counterparts, but more recent research has highlighted the unique differences and characteristics of young people engaging in sexual offending behaviors when compared to adults (Barra et al., 2018). Similarly, there has been little attention to whether there are differences from the perspective of victims in the experiences of sexual harm from adults compared to peers. Ever-changing societal norms about sexuality and what is considered developmentally appropriate and socially accepted, particularly for adolescents, continue to create uncertainty and disagreement about this phenomenon. Definitions are largely influenced by legal, social, and familial factors. Terms such as “developmentally appropriate” are also not easily defined or understood, particularly cross-culturally.

The broad term harmful sexual behavior (HSB) has been generally accepted across services and policymakers, particularly in Australia, New Zealand, and the United Kingdom. The term was designed to capture a range of sexual behaviors and experiences, including sexual offending, and it focuses on the behavior of concern rather than labeling the child or young person (Hackett et al., 2019). With the understanding of this phenomenon being in its relative infancy, further work is needed to generate knowledge and understanding about sexual harm inflicted by children and young people and its impacts. A shared conceptual understanding and definition of HSB would allow researchers, policymakers, legislators, service providers, and communities to understand, respond to, and prevent HSB.

## Objectives

This scoping review aims to understand the currently used definitions of HSB and how this phenomenon is described and understood. Researchers, practitioners, and policymakers have used multiple terms to describe sexual behaviors in children and young people. These include the following: problematic sexual behavior (PSB), reactive sexual behavior, sexual offending, HSB, sibling abuse, and peer-to-peer abuse. This scoping review aims to determine how these other terms fit within an overall definition of HSB, and CSA more broadly. The focus of this review was on children and young people (under 18 years) engaging in HSB toward other children, young people, and adults. Harm perpetrated by adults is beyond the scope of the present review.

## Method

For this scoping review, we used the five stages outlined by Arskey and O’Malley (2005): formulating a research question, identifying relevant papers through a systematic search of the evidence, screening the papers in line with inclusion and exclusion criteria, extracting data from the papers, and synthesizing findings using content analysis. This approach does not require the critical appraisal of papers but allows for the inclusion of both empirical studies and a broad range of gray literature including policy and practice documents. Due to the objectives of the scoping review, we also included research not published in scholarly peer-reviewed journals. We focused specifically on gray literature from Australia and the United Kingdom due to the term HSB being generally accepted across research, services, and policy in these jurisdictions.

The research questions that informed the scoping review were as follows:

1. How is HSB currently defined and described?
2. Are the terms “peer-to-peer abuse,” “sibling abuse,” etc., used to describe subsets of HSB?
  2.1. Are the terms used differently, depending on whether the focus is on the behaviors, the person engaging in the behavior, or the person subjected to the behavior?
  2.2. How do/does the definition(s) fit within the concepts of CSA and adolescent sexuality more broadly?
  2.3. Does the research literature distinguish between HSB and adult-perpetrated CSA?

3. Are the current definitions in use for HSB applied consistently across cultures, sectors, and stakeholder groups (education, psychology, policy, legal, and specialist sexual abuse services)?

Before we started the review, we registered a protocol via the Open Science Framework (OSF) (https://osf.io/qwc73/). OSF allows for the registration of protocols for scoping reviews.

### Eligibility Criteria

#### Inclusion Criteria

We screened only papers written in English, including both qualitative and quantitative studies of any design, as well as policy and practice documents such as government reports. To be eligible, papers could include the examination or discussion of victims, adult survivors, and children and young people who had engaged in HSB. Papers may have used other terms such as problem sexual behavior, sexual offending, or sibling sexual abuse. No limit was placed on the date of publication. We included papers only if they clearly defined sexual harm instigated by children and young people.

#### Exclusion Criteria

We excluded any studies only about harm perpetrated by adults. We excluded any sources that did not include a definition for HSB (or other similar term).

#### Search Strategy and Information Sources

Our systematic search strategy involved search terms to cover two concepts (see Table 1). We chose the first set of terms to focus search results on papers about children and young people. The second set of terms was designed to capture papers focused on HSB.

**Table 1.** Search Terms.

A. Child and Young People	B. HSB
Child* OR “young person” OR “adolescen*” OR student* OR teenage*	“harmful sexual* behavio*” OR HSB OR “problem* sexual* behavio*” OR PSB OR “reactive sexual* behavio*” OR “juvenile sex* offend*” OR “adolescent sex* offend*” OR “sibling* abuse” OR “peer* abuse” OR “sex* harm* behavio*” OR “sex* reactive behavio*”

*Note.* HSB = harmful sexual behaviors.

Six electronic databases were searched in May 2022: PsycINFO, MEDLINE, SocINDEX, Web of Science, ERIC, and Campbell Systematic Reviews. We selected these databases to gain a broad range of sources that would be covered in a range of settings and disciplines, including psychology, criminology, law, education, and social sciences. We employed the same search strategy for each database using the search terms set out in Table 1.

We also searched for gray literature from websites of key Australian and United Kingdom agencies, where there has been considerable policy and research focus on the problem of harmful sexual behavior (HSB) including Australian Institute of Family Studies, Australian Institute of Criminology, Australian Centre for Child Protection, Australian Government Royal Commission into Institutional Responses to CSA, Parenting Research Centre, Australia’s National Office of Child Safety, and the UK’s National Society for the Prevention of Cruelty to Children (NSPCC).

## Results

### Search Results

**Figure 1.**
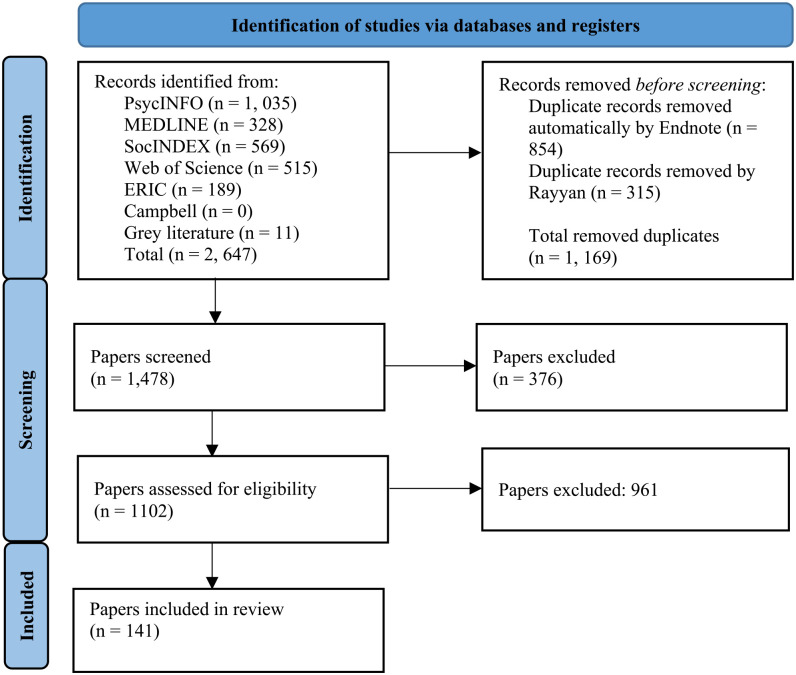
Preferred Reporting Items for Systematic Reviews and Meta-Analyses (PRISMA) Chart.

After systematic and targeted searching, we identified 1,478 papers for screening. All were double-blind screened by two independent reviewers in Rayyan (an online tool used to aid in the screening of studies for reviews). Any disagreements were resolved through discussion. A total of 141 papers were included for data extraction. Supplementary materials provide an overview of the included papers. Data extraction was completed by the first author in an Excel spreadsheet. A modified PRISMA flowchart is shown in Figure 1.

### Definitions

#### Definitions for Sexual Offending Behaviors

A large proportion (*N* *=* 59, over 40%) of the included papers included definitions for sexual offending behaviors. Terms used included juvenile sex offender, adolescent sex offender, sexual offending, illegal sexual behavior, and sexual abuse. The included papers did not use a consistent definition to describe this type of sexual behavior. Several of the excluded papers were also of this group but provided no definition, possibly due to the definition being seen as self-explanatory, as describing a legal-based definition of those under the age of the majority who have committed sexual offenses.

Defining sexual offending behaviors was generally limited to post-pubescent age groups, “those who commit such acts while between the age of puberty (12-14) and the age of the majority (18-20)” (Aljazireh, 1993, p. 423). This was largely reflective of the legislation for the relevant jurisdiction such as the age of criminal responsibility. Some papers extended the age of adolescence beyond 18 to reflect the developmental stage of those under the age of 21. For example, McKibbin et al. (2017) referenced Australian policy and research for their definition of children being zero to 12 years and young people being between the ages of 13 and 21 (McKibbin et al., 2017).

Gerardin and Thibaut (2004) defined sexual offending in adolescence as “youth who commits any sexual act with a person of any age against the victim’s will, or in an aggressive, exploitative, or threatening manner” (p. 79). Whereas other definitions simply focused on the illegal nature of the sexual behavior, rather than providing other descriptors (Bunston, 2000). Sexual offenses included both coercive and non-consensual acts and included both “hands-on” and “hands-off” offenses.

Although there were differences between included papers on the definitions used, largely the definitions for these terms included themes such as force, coercion, illegal behaviors, and non-consensual acts (Barbaree & Marshall, 2006; Bourke & Donohue, 1996; Dwyer & Boyd, 2009). Some also included themes of violence and aggression (Gerardin & Thibaut, 2004; Perry & Orchard, 1992).

Early definitions of sexual offending were largely influenced by Finkelhor’s (1986) definition of CSA (referenced in Craig et al., 2010), where an important feature was there being at least a 5-year age gap between perpetrator and victim. For example, Ryan and Colleagues’ (1987) definition of CSA perpetrated by an adolescent included the necessity of a 5-year age difference, as well as acts perpetrated against the victim’s will (referenced in Hastings et al., 1997). More recent definitions have removed the need for an age gap, noting that sexual harm can occur between children and young people of any age. Preference for understanding imbalances of power and use of force, coercion, or intimidation were more common in recent definitions.

Some authors theorized that it is possible to differentiate between adolescents who engage in sexual offending behaviors by the severity of their behaviors. Authors of several papers cited Aylwin and Colleagues’ (2000) definition that included six levels of severity increasing in the intrusiveness of sexual contact and levels of violence and force present. None of the included papers examined these severity groups but it was referenced as being used to assess treatment needs alongside a range of other risk assessment tools (Aebi et al., 2011; Barra et al., 2018). Some included papers that used groups or typologies to distinguish between different types of sexual offending behaviors based on the victim’s age or the relationship between the adolescent and the victim. For example, Craig and Colleagues (2010) drew on O’Brien’s (1991) four groups based on the relationship between the adolescent and the victim: (a) sibling incest, (b) child molester non-family, (c) non-child offender, and (d) mixed offender. In addition, differences between adolescents who sexually offended younger children with those who offended against same-aged peers or adults were explored (Keelan & Fremouw, 2013; Kjellgren et al., 2006). Across the included papers, it was clear that adolescents who engage in sexually offending behaviors are a heterogeneous group. Victim age, relationship, and the use of force were noted as important considerations for assessing treatment needs (Jensen et al., 2020; Lillard et al., 2020; Ryan & Otonichar, 2016; Ueda, 2017).

#### Definitions for PSB

A smaller proportion (*N* *=* 43, approximately 30%) of included papers examined PSBs in children under 12 years old (CEASE, 2016; El-Murr, 2017). Papers using this term often differentiated between the types of sexual behavior and developmental needs between pre- and post-pubescent children. This conceptual understanding may have been influenced by biological factors, such as the onset of puberty, social factors (such as social expectations and norms around sexuality and relationships), and legal influences (such as the age of criminal responsibility). Terms such as sexually reactive children, sexually acting out, and sexual behaviors of concern were also used. The themes identified were developmentally inappropriate behaviors, behaviors that involve secrecy and/or feelings of confusion and embarrassment, behaviors non-responsive to adult intervention, and behaviors that may be potentially harmful (Blomfield, 2018; Pelech et al., 2021; Pithers et al., 1998; Shawler et al. 2018; Silovsky et al., 2019).

One frequently referenced definition, developed by Chaffin et al. (2008), defined PSBs as:

children ages 12 and younger who initiate behaviors involving sexual body parts (i.e., genital, anus, buttocks, breasts) that are developmentally inappropriate, occurs at a greater frequency or at an earlier age than would be developmentally or culturally expected, become a preoccupation and/or reoccur after adult intervention, or are potentially harmful to themselves or others (use of force, coercion, or intimidation, cause physical injury or emotional distress, appear to be interfering with social development, involve children of substantially different ages). (p. 200)

However, Gray and Colleagues (1997, p. 271) noted that PSB may be “equivalent to criminal violation if performed by an adult,” noting that PSB may include quite serious and harmful sexual acts, even in pre-pubescent children. Imbalance in power and control were noted as important factors to consider in distinguishing between normal and PSB, regardless of the age group in consideration (Crisci et al., 1998; Offermann et al., 2008). For example, sexual behaviors that targeted a more vulnerable child were distressing, did not stop following parental intervention, or were persistent may be identified as problematic (Blomfield, 2018). Johnson’s (1991) early work in distinguishing between groups based on the nature of the sexual behavior, and Pithers et al.’s (1998) five considerations for problem sexual behavior in children informed several of the included papers in this area.

#### Definitions for HSB

HSB was the term most used in papers published by research from Australia and the United Kingdom and made up about 30% of the included papers (*N* *=* 44). This was likely influenced by Simon Hackett’s work for the NSPCC in the United Kingdom and the Australian Government’s Royal Commission into Institutional Responses to CSA adopting HSB as the term to describe harmful or abusive sexual behavior in children and young people (Australian Government, 2017; Hackett, 2014). Hackett’s (2014) definition and continuum of HSB was among one of the most frequently referenced:

sexual behaviours expressed by children and young people under the age of 18 years old that are developmentally inappropriate, may be harmful towards self or others, or be abusive towards another child, young person, or adult. (Hackett et al., 2019, derived from Hackett, 2014, p. 13)

In his continuum, Hackett (2011, 2014) distinguished between normal, inappropriate, problematic, abusive, and violent sexual behavior. In papers referencing Hackett’s (2014) definition and continuum (or similar variations), it was clear that the term HSB was designed to capture a large variety of sexual behaviors ranging from sexually abusive and violent behaviors such as rape, as well as potentially problematic or inappropriate behaviors in children and young people.

Although Hackett’s (2011, 2014) continuum and definition have been well-accepted and referenced across research, practice, and policy, there is still general disagreement about the understanding of “developmentally appropriate.” Other definitions for HSB focused instead on the imbalance of power and lack of consent while also indicating the harm caused to victims. For example, Calder and Colleagues (2001, p. 5) defined HSB as “any form of sexual activity with another individual, that they have powers over by virtue of age, emotional maturity, gender, physical strength, intellect, and where the victim in this relationship has suffered sexual exploitation and betrayal of trust” (referenced in Gibson, 2014). Hunter (2011) noted lack of consent (defined in their book as significant age differences or behavior that is manipulative or coercive) as the important factor in conceptualizing HSB.

The term “sexually abusive behaviors” was also used and generally referred to behaviors in adolescence, “sexually abusive behaviour applies to children and young people aged 10-17, defined by criminal law to be minors who have reached the minimum age of criminal responsibility” (El-Murr, 2017, p. 5). Overlaps in terms and themes were present—particularly relating to coercion, use of force, imbalance of power, lack of consent, intimidation, and aggression or violence—and were similar to the language used to describe sexual offending behaviors (ATSA, 2006; McCuish & Lussier, 2017). It was clear that this subset of behaviors could cause physical and emotional harm (Hackett et al., 2016). In more recent papers, terms such as sexually abusive behaviors were preferred compared to juvenile or adolescent sexual offending, largely to label the behavior of concern rather than labeling the young person (El-Murr, 2017). In some papers, these behaviors were linked with a broader understanding of CSA (Banks, 2014; Lane & Ryan, 2010; Malvaso et al., 2020; Righthand & Welch, 2004).

### Types or Subsets of HSB

Important types of sexual behavior were noted in some of the included papers. These included sibling sexual abuse, PSB across childhood and adolescence, and technology-assisted HSB. Unique definitions were presented in the papers for these and key findings pointed to distinct characteristics as compared to the broader conceptualization of HSB. We have also considered whether the perspective taken by the paper was on the child or young person instigating the sexual harm, on the person harmed, or both.

#### Victimization

One defining feature of the included papers was that most (approximately 97%) focused on the child or young person engaging in harmful, abusive, or problem sexual behavior. Very few (*N* *=* 4, less than 3%) included papers mentioned in their definition the child, young person, or adult who had been harmed or victimized. Of the included papers that focused entirely or in part on the victim-survivor, the chosen definitions were more commonly aimed at a broader definition of sexual abuse. For example, Kloppen and Colleagues (2016) referenced Butchart and Colleagues’ (2006, p. 19) definition of CSA that had a particular focus on the victim:

the involvement of a child in sexual activity that he or she does not fully comprehend, is unable to give informed consent to, or for which the child is not developmentally prepared, or else that violates the laws or social taboos of society. Children can be sexually abused by both adults and other children who are by virtue of their age or stage of development—in a position of responsibility, trust, or power over the victim.

In their study of educators’ understanding of sexual behaviors in children and adolescents, Ey and McInnes (2018) referenced Johnson’s (1988, p. 219) definition that considered children or young people to be perpetrators of sexual abuse when they:

(1) act in a sexual way with another child, (2) use force or coercion in order to obtain the participation of the other child or the victim was too young to realise they were being violated and did not resist the sexual behavior, or it was an offense such as exhibitionism, and (3) there was an age differential of at least two years, and (4) there was a pattern of sexually overt behavior in their history.

Themes of imbalance of power and the use of force or coercion were also present in Calder’s (1999, p. 2) definition of “child-on-child sexual abuse,” as children engaging in:

any form of sexual activity with another individual, that they have powers over by virtue of age, emotional maturity, gender, physical strength, intellect and where the victim in this relationship has suffered a sexual exploitation. (referenced in Yates, 2018)

Nearly all included papers noted that although the harms of sexual offenses (and other harmful or abusive sexual behaviors) committed by children and young people are comparable to those perpetrated by adults (Aljazireh, 1993; Bereiter & Mullen, 2012), young people are developmentally distinct and should be treated as a separate group from adult perpetrators of CSA (Barra et al., 2018). Davidson (1987) also noted an important distinction between sexual offending behaviors, or what they termed “sexually assaultive behavior,” and experimentation, innocent “sex-play,” and non-forcible sexual acts between non-related adolescents of a similar developmental stage.

#### PSB

It was clear from the included papers that separating pre-pubescent children from adolescents is important. PSBs in any age group were defined by some of the included papers as those that do not victimize others but may cause emotional distress or social rejection (Hackett et al., 2016; Meiksans & Bromfield, 2017). Many of the included papers acknowledged that these behaviors could result in physical, emotional, and social harms but the developmental differences and differences in types of behaviors between pre- and post-pubescent children were noteworthy and important.

With the introduction of Hackett’s definition of HSB (2014), the term PSB was included in their continuum of sexual behavior among children regardless of age. This acknowledged that young children (12 and under) may still engage in harmful and even abusive sexual behaviors and were not limited to the “problematic category” of sexual behavior. Similarly, this change also acknowledged that sexual behaviors in adolescents were not limited to just “normal” or “abusive” categories either.

#### Sibling Sexual Abuse

Publications on sibling sexual abuse, sibling incest, or other similar terms, specifically noted the harmful and abusive sexual behaviors that can be present in sibling relationships, even pre-puberty. Some included papers still pointed to age gaps in their definitions, “sexual behavior that is clearly exploitative in nature (i.e., where there is a significant age difference or where force, violence, or intimidation is employed)” (O’Brien, 1991, p. 75), or familial sexual abuse or intrafamilial sexual assault occurs when the age difference between siblings is greater than 5 years (Finkelhor, 1979; Craig et al., 2010). In these papers, they were more likely to use terms such as abuse and exploitation, suggesting that age gaps played a part in differentiating between sexual behaviors that were normal or problematic and those that were abusive or exploitative. Other papers noted that abusive or exploitative sexual behaviors may occur regardless of age gaps, with themes of imbalances of power and the use of force present. Since our search was conducted, a study by Marmor and Tener (2022) found unique typologies in cases of sibling sexual abuse. They concluded that it is important to consider different dynamics of sexual behavior.

#### Technology-Assisted HSB

Many of the definitions throughout the included papers were limited to contact sexual behaviors (involving touch or penetration) or non-contact behaviors such as exhibitionism and voyeurism but did not typically consider the use of technology. In fact, only three of the included papers specifically referenced technology in their definition (Armstrong, 2021; Lewis, 2018; McKibbin & Humphreys, 2019). Armstrong (2021, p. 3) provided a simple definition, “sexualised behavior carried out with the use of technology.” Lewis (2018) referenced Hollis and Belton’s (2017) definition of technology-assisted harmful sexual behavior as “one or more children engaging in sexual discussion or acts—using the internet and/or any image-creating/sharing or communication device—which is considered inappropriate and/or harmful given their age or stage of development” (p. 8). In a study examining the perceptions of Australian out-of-home care workers, McKibbin and Humphreys (2019) included technology as a consideration of how children and young people may experience sexual exploitation by referencing Beckett and Colleagues (2017) definition:

a form of child abuse that occurs when an individual or group takes advantage of an imbalance of power to coerce, manipulate or deceive a child or young person under the age of 18 into sexual activity (a) in exchange for something the victim needs or wants and/or (b) for the financial advantage or increased status of the perpetrator or facilitator. The victim may have been sexually exploited even if the sexual activity appears consensual and does not always involve physical contact, as it can also occur through the use of technology. (p. 7)

### Key Findings

Across the included studies, regardless of the terminology used, it was clear that HSB is a significant issue facing families, schools, and our criminal justice and child protection systems (Baker et al., 2001; Banks, 2014; Draugedalen, 2021; Ey & McInnes, 2018; Firmin, 2020; Lloyd, 2019; McInnes & Ey, 2020; McKibbin et al., 2022; Waters et al., 2021; Yates, 2018). Definitions were applied inconsistently across stakeholder groups, and the use of terminology was different depending on whether the focus was on the victim-survivor, or the child or young people causing the harm. There were clear subsets identified, but these were also defined inconsistently across the literature. Across the included papers, there was a clear distinction between (a) normal sexual development (Davidson, 1987), (b) sexual behaviors that are harmful or abusive, and (c) adult-perpetrated CSA.

Trauma and other adverse childhood events were consistently identified in the histories of those presenting with HSB, PSB, and sexual offending behaviors, regardless of the severity of the behavior, the setting, or the age group of interest. Experiences such as sexual abuse victimization, experiences of alternative care (out-of-home care), harsh or neglectful parenting practices, exposure to violence, parental arrest, and socioeconomic disadvantage were identified across several papers (Aljazireh, 1993; Blomfield, 2018; Delago et al., 2020; Felizzi, 2015; Gray et al., 1997; Hawkes, 2011; Lévesque et al., 2010; Szanto et al., 2012).

Although many researchers identified trauma and adversity in the history of children and young people presenting with HSBs, it was apparent that they are a heterogeneous group (Bourke & Donohue, 1996; Johnson, 2002). Early intervention was key, with youths exhibiting PSBs in early childhood having a higher re-offense rate than those who first exhibited HSBs in middle childhood or adolescence (Grossi et al., 2012). Parental involvement, social supports, adequate risk assessment, sex education, and involving young people in the development of safety plans were identified as key to intervention (Apsche & Ward, 2003; Barra et al., 2018; Bastos et al., 2021; Clionsky & N’Zi, 2019; Gibson, 2014; Marriage et al., 2017; McKibbin, 2017; Prisco, 2015; Shawler et al., 2018; Stith & Bischof, 1996).

## Discussion

The purpose of our scoping review was to better understand the definitions used in existing research, policy, and practice for HSB in children and young people. It was clear that how HSBs are interpreted and defined is inconsistent and has been influenced by time and cultural values. This has made comparison between studies challenging and likely means that we do not have an accurate assessment of the prevalence, nature, etiology, or treatment needs for this population. It is important to note that the present review is limited to definitions and conceptual understandings present in the included papers. Several excluded papers included an examination of key concepts but failed to include a specific definition of the sexual behavior of interest. As well as the international peer-reviewed literature search, we supplemented this with a particular focus on gray literature from agencies in Australia, New Zealand, and the United Kingdom given their similar approach to terminology. Therefore, findings are particularly relevant to these jurisdictions. Further exploration is needed for this concept in other contexts, including in non-Western cultures.

Hackett’s (2011, 2014) continuum and definition of HSB were commonly cited across a range of stakeholder groups including policy, practice, education, out-of-home care, and child welfare. Consideration of the developmental context in differentiating between normal, problematic, and harmful or abusive sexual behavior, and the subsequent harms that can be caused was highlighted throughout his work (Hackett, 2014; Hackett et al., 2016, 2019). Although Hackett (2014) suggested HSB as a broad umbrella term for a range of sexual behaviors in children and young people, he noted that there is merit in using different terminology to describe subsets of the proposed continuum. In the adult-perpetrated sexual abuse literature, there is precedence for using distinct terms to describe different types of sexual harm and abuse. For example, there are distinctions made between workplace sexual harassment, rape, and sexual abuse in the context of interpersonal relationships. This approach creates clarity and allows for research and practice that addresses the unique needs of each type of sexual behavior.

We suggest that sexual abuse, sexual offending behavior, problem sexual behavior, sibling sexual abuse, and technology-assisted HSB are examples of important types of HSBs in children and young people. There were key differences identified relating to etiology and treatment needs depending on the relationship between the victim and instigating young person (e.g., victims that are siblings, peers, or other), victim age, use of force, and type of sexual behavior (Aylwin et al., 2000; Butz & Spaccarelli, 1999; Craig et al., 2010; Jensen et al., 2020; Keelan & Fremouv, 2013; Kjellgren et al., 2006; Lillard et al., 2020; Marriage et al., 2017; Ueda, 2017). For example, adolescents who had offended younger children were found to be more likely to have experienced trauma earlier in life, have multiple victims, have lower self-esteem, have more internalizing behavior problems, and be indiscriminate of relationship to and gender of the victim, compared to those who had offended against peers or adults (Jensen et al., 2020; Lillard et al., 2020; Ueda, 2017). Adolescents who used force, including verbally and physically coercive sexual behaviors, were more likely to display misogynistic fantasies, sexual compulsivity, and hypermasculinity, compared to those who did not use force as part of their sexual offending behaviors (Johnson & Knight, 2000). Furthermore, an analysis of Australian cases of problem sexual behavior in children revealed five distinct groups (sexually aggressive, non-symptomatic, highly traumatized, rule breaker, and abuse reactive) based on underlying factors that contributed to the sexual behaviors, rather than the nature of the sexual behavior itself. These factors included maltreatment history, diagnosis, and aggression (Pithers et al., 1998). These findings support the notion that using a wide range of terminology to conceptualize subtypes of this phenomenon is important as it informs best practice and prevention.

Most of the included papers had a clear focus on the child or young person exhibiting the behavior. Importantly, of those that included a focus on victim-survivors, it was more likely that the chosen definition would reflect a broader conceptualization of CSA. This reflected an important distinction in understanding of this phenomenon based on the perspective taken. Perhaps a focus on victim-survivors lends itself to an understanding of the abusive nature of certain types of HSB and the comparable impacts these behaviors have to adult-perpetrated sexual abuse, thus using terms such as sexual abuse. However, in cases involving children and young people, there is not always a clear distinction between perpetrator and victim. Similarly, key findings from workers’ perceptions in Australia found that children and young people often did not identify themselves as victims in cases of child sexual exploitation (McKibbin & Humphreys, 2019). These findings highlight key issues in conceptualizing sexual harm inflicted by children and young people, particularly in the digital world.

Although not an included paper in this review, Mathews and Collin-Vézina’s (2017) conceptual model of CSA suggests four factors that must be present in order for an act or experience to be conceptualized as CSA the victim must be a child either developmentally or considered by the law or society norms; true consent must be absent; the acts must be sexual; and the acts must constitute abuse. Relationships where there is an imbalance of power, a position of inequality, and exploitation of vulnerability may constitute abuse. In their conceptual model of CSA, the age difference between victim and perpetrator, and harm were not preconditions to an act being considered sexually abusive, noting that both adolescents and adults may perpetrate CSA (Mathews & Collin-Vézina, 2017).

There was a consensus that although certain types of HSB are clearly abusive in nature to victim-survivors, how children and young people who engage in these behaviors are described is important. More recent publications were largely in favor of terms labeling the behavior rather than labeling the child or young person. For example, labels such as “adolescent sex offender” are inappropriate and stigmatizing. Reducing the stigma associated with labeling a child or young person as an “offender” while also acknowledging the harm caused and the serious nature of the behaviors is important and should be reflected in the terminology used (Barry & Harris, 2019; Blomfield, 2018; El-Murr, 2017; Hackett, 2014).

### Critical Findings

A large majority of the papers in our review relied on samples from correctional, criminal justice, or child protection settings. It is likely that this approach represents only the most serious end of sexual offending behaviors in adolescence, only one subset of this phenomenon. With papers focusing on strict legal definitions for sexual offending behaviors, a large proportion of both victims and instigating children and young people are undetected (Gewirtz-Meydan & Finkelhor, 2020), and victims are less likely to identify themselves as having been harmed (McKibbin & Humphreys, 2019), many children and young people are less likely to receive intervention or participate in empirical research. Most papers were also based on English-speaking populations, with few including consideration of diverse groups. These issues highlight a significant gap in the research base and understanding of a broad range of HSB subtypes in children and young people.

### Implications for Practice, Policy, and Research

Clear and operational definitions for important subsets of HSB across sectors would allow for clarity in conceptualizing and preventing this issue. Clearly defined terms are also essential for service providers, parents and carers, and other professionals to be better equipped to identify problems, harmful, or abusive sexual behaviors, respond appropriately, and prevent further harm. Relying solely on one term to describe a range of sexual behaviors present in children and young people limits our understanding of the different developmental pathways and treatment needs, and implies similarities between groups that are not present. Research is also needed on diverse groups presenting with different subsets of this phenomenon in different settings. It is not clear if current findings are generalizable across cultures or settings (i.e., outside of criminal justice or child protection settings).

Findings point to key factors that contribute to risk, particularly child maltreatment and adversity (Aljazireh, 1993; Blomfield, 2018; Delago et al., 2020; Felizzi, 2015; Gray et al., 1997; Hawkes et al., 2000; Lévesque et al., 2010; Szanto et al., 2019). These factors can be targeted from a broad public health approach model. Parents, teachers, and other youth-serving organizations are key targets for prevention. This can include parenting supports to target harsh parenting practices and reduce the risk of child abuse and neglect, socioeconomic and social supports, training and education for teachers and other workers, and adequate sex education. Parental involvement and assessment are pivotal in improving outcomes (Clinosky & N’Zi, 2019; Kloppen et al., 2016; Kulesz & Wyse, 2007). We need flexibility in our approaches to this issue. This allows for responses and interventions that target the individual needs of the child or young person, and result in better outcomes. This might include family-based interventions, such as Multi-Systemic Therapy (MST-HSB), where insecure attachment, poor parental monitoring and supervision, and abuse and neglect have played a role (Fonagy et al., 2015; Pourliakas et al., 2016).

We suggest that although HSB may be a useful umbrella term adopted by researchers, policymakers, and practitioners alike, it is important to also acknowledge the large variance in behaviors, treatment needs, etiology, and harms present across the subtypes of HSB. Using terms such as CSA may be appropriate in the context of working with victim-survivors of HSB, giving acknowledgment to the comparable harms and impacts as with adult-perpetrated CSA. Using terms for other subsets of HSB such as sexual abuse, sibling sexual abuse, technology-assisted HSB, sexually abusive behavior, and problem sexual behavior gives more context to the behavior of concern and may be helpful for informing further research, prevention, and best practice approaches.

## Supplemental Material

sj-docx-1-tva-10.1177_15248380231218294 – Supplemental material for Scoping Review of the Definitions Used to Describe and Understand Harmful Sexual Behaviors in Children and Young People Supplemental material, sj-docx-1-tva-10.1177_15248380231218294 for Scoping Review of the Definitions Used to Describe and Understand Harmful Sexual Behaviors in Children and Young People by Gabrielle R. Hunt, Daryl J. Higgins, Megan L. Willis and Lottie Harris in Trauma, Violence, & Abuse
