# Supplementary material for: Scoping Review of the Definitions Used to Describe and Understand Harmful Sexual Behaviors in Children and Young People
Source: Trauma Violence Abuse. 2023 Dec 28;25(4):2569–83. doi: 10.1177/15248380231218294 (PMC11370191; doi:10.1177/15248380231218294)
Supplement: sj-docx-1-tva-10.1177_15248380231218294 – Supplemental material for Scoping Review of the Definitions Used to Describe and Understand Harmful Sexual Behaviors in Children and Young People [file sj-docx-1-tva-10.1177_15248380231218294.docx]

**Table 2. Included Papers that Used Terms Related to Sexual Offending Behaviours**

| **Author(s)** | **Year** | **Region** | **Paper Type/Study Design** | **Population** | **Key Findings** | **Definition** |
| --- | --- | --- | --- | --- | --- | --- |
| Aebi et al. | 2011 | Switzerland | Cross-sectional | Juveniles convicted of a sexual crime. | Judicial files were used to assess the utility of risk of recidivism assessment tools. J-SOAP-II and SOS seem useful for assessing risk but further risk assessment seems necessary. | 6 levels of severity based on type of offending behaviour (Aylwin et al., 2000). |
| Aljazireh | 1993 | USA | Critical review | Male adolescent sexual offenders. | Reviewed the research on correlates of sexual offending by male adolescents. There is strong evidence that supports the influence of early childhood victimisation and previous delinquency. | Adolescents who commit such acts while between the age of puberty (12 to 14) and the age of the majority (18 to 20). |
| Apsche et al. | 2008 | USA | Randomised control trial | Families in Children and Youth Services. | Compared treatment outcomes between MDT and treatment as usual, where MDT was found to be more effective in improving family relationships and reducing family disharmony. | Fondling, fellacio, deviant sexual intercourse, and any other sexual behaviour determined by the Children and Youth and/or the court as illegal or problematic, or a charged defence. |
| Bastos et al. | 2021 | Brazil | Cross-sectional | Male adolescent sexual offenders. | Aimed at understanding the key characteristics of adolescent sexual offenders in Brazil. Most victims were extrafamilial and between the ages of 10 and 14. Social context and family relationships were key for intervention. | Any act or conduct aimed at the sexual satisfaction of an adult or adolescent with a significant age difference from the victim, which may or may not include physical contact or penetration. |
| Barbaree & Marshall | 2006 | Canada | Book chapter | Juvenile sex offenders. | Discussion of definitions and laws in Canada and US. | Sex offenders are persons who have been convicted in a criminal court of a sexual crime. Sexual crimes include those that are nominally sexual (e.g., sexual assault, rape) and crimes that have some sexual intent or component (e.g., murder or attempted murder during the commission of a rape, simple assault pled down from rape). |
| Barra et al. | 2018 | Switzerland | Cross-sectional | Juvenile sex offenders. | Risk assessments are useful but must not be relied on solely. For example, elevated offence severity and burden of ACEs impeded the predictive accuracy of the J-SOAP-II and VRAG-II, particularly in case of sexual recidivism. | 6 levels of severity based on type of offending behaviour (Aylwin et al., 2000). |
| Becker & Johnson | 2001 | USA | Book chapter | Juvenile sex offenders. | Provides an overview of treatment considerations and approaches for juvenile sex offenders. | An individual who has committed overt sexual acts that are contrary to existing sexual mores in their society (Gebhard et al., 1964). |
| Bereiter & Mullen | 2012 | USA | Book chapter | Juvenile sex offenders. | Provides an overview of the literature on treatment approaches for juvenile sex offenders. | Someone who is convicted of a sexual offense and is considered by the court to be old enough to be held criminally responsible but not old enough for full adult criminal sanctions (Barbaree & Marshall, 2006). |
| Bourke & Donohue | 1996 | USA | Literature review | Juvenile sex offenders. | JSOs are a heterogeneous group which makes classification and the ability to generalise findings difficult. | Any forced or coercive sexual contact, substantial threats of such contact with a much younger child, or any non-consensual sexual behaviour that violates conventional standards (Smith & Monastersky, 1986).  3 major types - (1) passive or "hands-off" offences (e.g. voyeurism, obsence phone calls, stealing underwear, exhibitionism; (2) "hands-on" offences involving some degree of force, aggression or coercion (e.g. rape, sexual assault, fondling, and attempted rape); (3) pedophiliac offences, which occur when an individual acts out sexually against victims 4 or more years younger than themselves |
| Bromberg & O'Donohue | 2013 | UK | Book | Children and adolescents. | Book on sexuality in children and adolescents, including chapters on problem sexual behaviour and sexual offending. | Hands-on offences (e.g. molestation) and hands-off offences (e.g. exhibitionism), with varying degrees of coercion against victims of all ages. |
| Bunston | 2000 | Australia | Review | Adolescent sexual offenders. | Review of definition and profile of adolescent sexual offender and assessment and treatment approaches. | Adolescent who commits sex offences, aged between twelve and eighteen years, who engages in sexual behaviour deemed by society to be illegal. Sexual offences comprise both coercive and non-consensual sexual acts (Perry & Orchard, 1992). |
| Burke | 2001 | USA | Cross-sectional | Adolescent sexual offenders. | Adolescent sexual offenders scored significantly lower overall on empathy compared to non-sexual offenders. | Use of coercion or force, sexualised interactions which are age-inappropriate for the partner, and partners who are not peers (Vizard et al., 1995). |
| Butz & Spaccarelli | 1999 | USA | Cross-sectional | Juvenile sex offenders. | 3 groups were defined (rapists, nonrapists, and deniers) based on the use of force. | 4 level taxonomy based on type and severity of sexual behaviour (Johnson, 1993). |
| Cashwell & Caruso | 1997 | USA | Review | Adolescent sex offenders. | Mental health counsellors should have skills to respond to adolescent sexual offenders, due to this group being responsible for a substantial proportion of sexual victimisation. | 4 groups based on 'appropriateness' of sexual behaviours and key factors for the instigating child (Johnson & Feldmeth, 1993). |
| Clionsky & N'Zi | 2019 | USA | Review | Adolescents with autism spectrum disorder who engage in HSB. | Developmental and sexual assessment is crucial to assessment and treatment. | Adolescents with illegal sexual behaviours are typically between 13 and 18 whose sexual behaviour breaks the laws established by their current jurisdiction (Silovsky & Bonner, 2003). |
| Craig et al. | 2010 | UK | Book chapter | Adolescents sexual offenders with intellectual disabilities. | Provides an overview of assessment and treatment approaches for adolescent sex offenders with intellectual disabilities. | Familial sexual abuse/intrafamilial sexual assault has been described as exploitation where the age difference between the siblings is greater than 5 years (De Long, 1989; Finkelhor, 1979).  4 groups, (1) sibling incest, (2) child molester non-family, (3) non-child offender, (4) mixed offender (O’Brien, 1991). |
| Davidson | 1987 | USA | Review | Juvenile sex offenders. | Provides a discussion of legal responses to juvenile sex offenders. | JSO as a term to describe sexually assaultive behaviour (e.g. rape, incest, forcible sexual contact). |
| Dwyer & Boyd | 2009 | USA | Review | Adolescent sex offenders. | Sex education identified as an important component of treatment. | Male between the ages of 12 and 18 who has engaged in sexual activity involving another person, who did not provide consent literally or based on being under the legal age of consent. |
| Ey & McInnes | 2018 | Australia | Qualitative | Educators. | 40.8% of educators had observed children displaying problem sexual behaviours. | Sexual acts perpetrated on another without consent, without equality, or as a result of coercion (Shaw et al., 2000). Children considered perpetrators of sexual abuse when they (1) act in a sexual way with another child, (2) used force or coercion in order to obtain the participation of the other child or the victim was too young to realise they were being violated and did not resist the sexual behaviour, or it was an offense such as exhibitionism, and (3) there was an age differential of at least two years, and (4) there was a pattern of sexually overt behaviour in their history (Johnson, 1988). |
| Felizzi | 2015 | USA | Cross-sectional | 502 juvenile male sexual offenders and nonsexual offenders. | Multiple relocations or homelessness, children placed out of home, slapping or punching in the home, and sexual abuse victimisation were most highly related to sexual offending in juveniles. | Any sexual interaction with a person of any age that is perpetrated (a) against the victim's will, (b) without consent, or (c) in an aggressive, exploitative, manipulative, or threatening manner (Ryan & Lane, 1997). |
| Gerardin & Thibaut | 2004 | France | Review | Juvenile sex offenders. | Treatment should include behavioural therapy, family therapy and psychosocial intervention. | Youth who commits any sexual act with a person of any age against the victim's will, or in an aggressive, exploitative, or threatening manner. |
| Grant | 2000 | Australia | Review | Adolescent sex offenders. | Early intervention is more effective in the treatment of this group. | Young people aged 18 or under who engage in sexually assaultive behaviour (for example, rape or forcible sexual contact; Davidson, 1987). |
| Hastings et al. | 1997 | USA | Cross-sectional | Adolescent sex offenders and adolescents with conduct disorder. | Conduct disordered youth had higher socialised aggression and avoidance coping than adolescent sex offenders. | Sexual abuse against children perpetrated by an adolescent - (1) the victim at least 5 years younger than the perpetrator and under 16 years of age, (2) the perpetrator engaged in a sexual act involving genital stimulation, oral sex, or penetration against the victim's will (Ryan et al., 1987). |
| Johnson & Knight | 2000 | USA | Cross-sectional | Juvenile sex offenders. | Sexual compulsivity and hypermasculinity significantly discriminate verbally and physically coercive juvenile offenders from those that do not report using force. | Individuals age 28 or younger who commit a sexual offense against a victim of any age. |
| Keelan & Fremouw | 2013 | USA | Review | Adolescent sex offenders. | Examined differences between child and peer-adult offenders. Inconsistent findings and definitions were present, however this may be a helpful categorisation for understanding treatment needs. | Any sexual interaction with a person of any age that is perpetrated (a) against the victim's will, (b) without consent, or (c) in an aggressive, exploitative, manipulative, or threatening manner (Ryan, 2010). |
| Keogh & Ruszczynski | 2018 | Australia | Book | Juvenile sex offenders. | Book chapter on juvenile sex offenders. | Non-consensual sexual behaviour involving another person and encompassing force and/or manipulation (Ryan et al., 2010). |
| Kjellgren et al. | 2006 | Sweden | Cross-sectional | Adolescent sex offenders. | 46% had abused at least one child younger that 12, whereas the rest had abused peer or adult victims. | Sexual abuse occurs when a person is subjected to a sexual act against his or her will. This could include physical contact ("hands-on"), or sexual harassment without physical contact with the victim ("hands-off"). |
| Kloppen et al. | 2016 | Norway | Literature review | Child sexual abuse victims. | Increased risk of abuse from early adolescence, with peers being the largest group of perpetrators. Targeting peer abuse seems to be vital in the prevention of child sexual abuse. | The involvement of a child in sexual activity that he or she does not fully comprehend, is unable to give informed consent to, or for which the child is not developmentally prepared, or else that violates the laws or social taboos of society. Children can be sexually abused by both adults and other children who are - by virtue of their age or stage of development - in a position of responsibility, trust, or power over the victim (Butchart et al., 2006). |
| Kraemer et al. | 1998 | USA | Cross-sectional | Juvenile child molesters. | Younger juveniles may be more amenable to treatment. Impulsivity seems to be an important factor in treatment. | Offenders who were at least 3 years older than their victims and who had used either bribery, coercion, manipulation, or a combination of these factors, rather than physical force, to sexually molest their victims. |
| Lakey | 1995 | UK | Review | Male adolescent sexual offenders. | Multiple factors contribute to the development of sexual offending behaviour. | Youth ranging from puberty to the age of legal majority who commits any sexual interaction with a person of any age against the victim's will, without consent, or in an aggressive, exploitative, or threatening manner (Scavo & Buchanan, 1989). |
| Lane & Ryan | 2010 | USA | Book | Juvenile sex offenders. | Provides information on causes, consequences and correction of juvenile sex offenders. | Any sexual interaction with a person of any age that is perpetrated (a) against the victim's will, (b) without consent, or (c) in an aggressive, exploitative, manipulative, or threatening manner. |
| Leroux et al. | 2020 | Canada | Cross-sectional | Adolescent sex offenders. | Sexual victimisation along with engaging in risky sexual behaviours may help explain pathway to sexual offending. | Sexual activities that have been empirically demonstrated to increase the likelihood of negative health (e.g. STI) and psychosocial (e.g. sexual victimisation) outcomes (Lalor & McElvaney, 2010). |
| Lewis et al. | 1979 | USA | Cross-sectional | Sexually assaultive juveniles and violent juveniles. | Incarcerated sample had higher rates of recidivism and violent behaviours than previous studies (may be due to their focus on sexual deviance in general). | Having been found guilty of the act of sexual assault by the juvenile court, sentenced to a correctional school and placed by judges or school administrators in secure custody because of the nature of the acts committed. |
| Lillard et al. | 2020 | USA | Cross-sectional | Juvenile sex offenders. | Peer offenders had more severe sexual offences, prior status/nonviolent charges and issues with sexual functioning. Mixed began offending at a younger age and were indiscriminate in gender and relationship of the victim. | Any sexual interaction with a person of any age that is perpetrated (a) against the victim's will, (b) without consent, or (c) in an aggressive, exploitative, manipulative, or threatening manner (Ryan et al., 2012). |
| Margari et al. | 2015 | Italy | Cross-sectional | Juvenile sex offenders. | Juvenile sex offenders differed from non-sex offenders on some domains such as living in single-parent homes, academic failure and previous sexual intercourse. | Spectrum of behaviours with physical contact (frottage, fondling, groping and all aggressive acts with penetration) and without physical contact (exhibitionism, voyeurism, obsence phone calls) (Shaw, 1999). |
| Margolin | 1983 | USA | Review | Adolescent sexual offenders. | Reviews the nature, responses and treatment of adolescent sex offenders. | "Hands-on" offense is a coercive sexual act which involves direct physical contact between the offender and the victim, most commonly encompassing rape and child molestation. |
| McCuish & Lussier | 2017 | Canada | Review | Juvenile sex offenders. | Suggested that there are limitations to typology approaches. A focus on what happens at the time of the offense, rather than what lead up to that moment and the time after may be more relevant. | (a) violates a person's right to autonomy over decisions involving direct and indirect interactions of a sexual nature, (b) involves a sexual interaction with someone too young to consent, (c) involves a sexual interaction between two individuals of discrepant ages, or (d) involves a perpetrator acting in a position of authority. |
| Messerschmidt | 2011 | USA | Qualitative | Female adolescent sex offenders who have experienced bullying. | Culture plays an important role in understanding this phenomenon. | By manipulation, engaged in sexual contact (e.g. fondling) with boys legally unable to give informed consent. |
| Miccio-Fonseca | 1996 | USA | Cross-sectional | Adult and adolescent sex offenders and victims. | The 4 groups differed significantly across a number of measures, e.g. offenders/victims group had a higher percentage of psychological difficulties, being psychiatrically hospitalised, and being in treatment. | A person who has admitted to, or been convicted of, a sex crime or has encountered legal difficulties because of his/her sexual habits. |
| Okami | 1992 | USA | Literature review | Child perpetrators of sexual abuse | Concludes that much of the work on child perpetrated sexual abuse has been influenced by moral panic whereby adults overreact to voluntary peer sexual interactions. | Child perpetrator criteria for inclusion are: (1) they had acted in a sexual way with another child, (2) they had used force or coercion in order to obtain the participation of the other child, or the victim was too young to realise they were being violated and did not resist the sexual behaviour, or it was an offense such as exhibitionism, (3) there was generally an age differential of at least two years for children 9 years or older. The age differential in younger children may be less. It was not always the older child who was the initiator of the sexual behaviours, (4) there was a pattern of sexually overt behaviour in their history (Johnson, 1988). |
| Oliver et al. | 1993 | USA | Cross-sectional | Adolescent sex offenders, and non-sexual offending adolescents | Adolescent sex offenders generally displayed the least deviant personality and historical characteristics. Adolescents may be more amenable to treatment than their adult counterparts. | Any sexual behaviour that is coercive (i.e. rape, child molestation, gross sexual imposition) or involves individuals who are unable to give informed consent. |
| Openshaw et al. | 1993 | USA | Literature review | Youthful sexual offenders | Review of the research on youthful sexual offenders. | Preadult male or female who instigates sexual or assaultive sexual interaction with either a nonconsenting partner or a child too young to understand the sexual behaviour being consented to. |
| Prisco | 2015 | USA | Review | Parents | Supervision and treatment are more effective if parents or guardians are involved. | Individuals at or below the maximum age of juvenile court jurisdiction who commits a sex offense against another individual, such as rape, exhibitionism (Rothchild, supra note 10, at 723). |
| Rasmussen & Dibble | 1989 | USA | Review | Juvenile sex offenders | Report on juvenile sex offenders in Utah. | (a) any juvenile below the age of 18 who has committed a sexual act defined by the Utah Criminal Code Annotated, (b) the act might be defined as a sexual offense if it meets any one of the following: power differential (age difference, larger physical size, greater mental capacity, greater physical capacity), role differential, predatory patterns, elements of coercion, (c) the sexual act might include any of the following behaviours: fondling, frottage, digital penetration, oral copulation, object insertion, penile penetration, "hands-off" offences (voyeurism, exhibitionism, and/or obscene phone calls) |
| Ricks et al. | 2015 | USA | Book | Adolescent sex offenders | Book on adolescent sexual offending. | Any sexual contact which involves coercion, manipulation of power, or is committed against individuals who are unable to give informed consent. |
| Righthand & Welch | 2004 | USA | Literature review | Juvenile sex offenders | Use of language when talking about this group is important, use of behaviour first language (e.g. sexually abusive behaviour) is preferred. | Sexual misconduct including non-contact sexual behaviours (such as exhibitionism and voyeurism) and penetrative acts. |
| Runyon et al | 2006 | USA | Book | Children who have experienced maltreatment. | Book chapter on aetiology and surveillance of different types of child maltreatment. | Exploitation, involvement, or exposure of children to age-inappropriate sexual behaviour by older or "more powerful" peers of adults for purposes of sexual gratification. |
| Ryan et al. | 2012 | USA | Book | Juvenile sex offenders. | Book chapter on juvenile sex offenders. | Purposeful sexual act committed against another person, which may include physical, verbal, or other forms of coercion or manipulation (American Psychiatric Association, 1999). |
| Ryan & Otonichar | 2016 | USA | Literature review | Juvenile sex offenders. | Individualised evaluation is critical in order to make appropriate disposition and treatment recommendations that take into account risk of recidivism. Risk factors for reoffending include multiple prior sexual offences, selection of a stranger, and deviant sexual interests. | Youths between the ages of 12 and 18 who have been charged with or convicted of a sexual crime, or have engaged in an act that could be officially charged as a sexual crime, or have committed any sexual act with a person of any age in an aggressive, threatening, or exploitative manner (Gerardin & Thibaut, 2004). |
| Scavo & Buchanan | 1989 | USA | Case study | Male adolescent sexual offenders. | A multi-disciplinary approach to integrate youth offender's group treatment goals into all areas of residential program is important. | Youth ranging from puberty to the age of legal majority who commits any sexual interaction with a person of any age against the victim's will, without consent, or in an aggressive, exploitative, or threatening manner. |
| Schmidt et al. | 2012 | USA | Book | Adolescents with illegal sexual behaviour. | Book chapter on understanding and treating adolescents with illegal sexual behaviour. | Girls and boys from age 13 to 18 who have committed a sexual act that is illegal in the jurisdiction where the behaviour occurred. |
| Schnitzer et al. | 2020 | UK | Systematic literature review | Adolescent sex offenders. | 6 case studies reviewed, interventions included detailed assessments, staff training, peer support, medication, and adapted CBT. Overall quality of studies was low. | Minor who commits a sexual act with a person of any age when the offence is against the victim's will, without consent, or perpetrated in an aggressive, exploitative, or threatening manner (Ryan et al., 2010). |
| Sneddon et al. | 2020 | UK | Cochrane review | Young people with HSB. | It was uncertain whether CBT reduces HSB in male adolescents compared to other treatments. All studies had insufficient detail. | Person aged between 10 and 18 years old who has been reprimanded, warned, or convicted of a sexual offence, or who has received civil measures for their sexual offending. |
| Sperry & Gilbert | 2005 | USA | Case review | Children with peer sexual behaviours. | Compared to abuse by peers, abuse perpetrated by adolescents/adults was more intrusive and intrafamilial. Both groups rated their experience as equally negative, and reported equally pervasive outcomes. Participants abused by peers anticipated less support from both parents and more anger from their mothers. | Peer sexual abuse as any forced or unwanted sexual experiences between individuals less than 5 years apart in age (Finkelhor, 1986). |
| Stith & Bischof | 1996 | USA | Book chapter | Adolescent sex offenders. | A lack of positive overall communication with parents may be a factor in the development of sexual offending behaviour. | Youth from puberty to the legal age of majority who commits any sexual act with a person of any age, against the victim's will, without consent, or in an aggressive, exploitative or threatening manner (Ryan, 1986). |
| Timms & Goreczny | 2002 | USA | Literature review | Adolescent sex offenders with mental retardation | Social deficits in individuals with mental retardation may contribute to aberrant sexual behaviour among adolescents. | A minor who commits any sexual act with a person of any age when the offense is against the victim’s will, without consent, or perpetrated in an aggressive, exploitative, or threatening manner (Ryan, 1991). |
| Thibaut et al. | 2016 | France, Canada, Germany, Chile, Belgium | Guidelines | Adolescent sex offenders with paraphilic disorders | Adolescents were in various stages of puberty and development which may limit use of pharmacological agents in comparison to adults. | Youths between the ages of 12 and 18 who have been charged with or convicted of a sexual crime, or have engaged in an act that could be officially charged as a sexual crime, or have committed any sexual act with a person of any age in an aggressive, exploitative or threatening manner (Geradin & Thibaut, 2004). |
| Ueda | 2017 | Canada | Review | Juvenile sex offenders | Adolescents who had engaged in sexual offending behaviours against younger children were more likely to be submissive, have low self-esteem, and have internalising behaviour problems compared to those who offend against peers or adults. | 12-18 year olds who commit sexual offences. |
| Valdivia-Salas et al. | 2021 | Spain | Cross-sectional | Spanish adolescents who had at least one romantic relationship within the last year. | Occasional and frequent teen dating violence (physical and emotional) was more frequent in girls than in boys, and that personal distress functioned as a partial mediator. | Teen dating violence may be psychological (e.g. emotional manipulation), physical (e.g. shoving, slapping, kicking), and/or sexual (e.g. forced sexual activity; Saltzman et al., 2002). |

**Table 3. Included Papers that Used Terms Related to Problem Sexual Behaviour**

| **Author(s)** | **Year** | **Region** | **Paper Type/Study Design** | **Population** | **Key Findings** | **Definition** |
| --- | --- | --- | --- | --- | --- | --- |
| Baker et al. | 2002 | USA | Cross-sectional | Children in the NY child welfare system | Most agencies reported PSB as a significant problem for staff and families. | Sexual behaviour that caused a problem for the youth, other children, or adults (Ryan & Blum, 1993). |
| Blomfield | 2018 | Australia | Case studies | Children engaged in problem sexual behaviours | PSBs frequently occur in the context of complex factors including inadequate supervision, abuse, family instability, and dysfunction. | Sexual behaviours may be considered problematic if they target a more vulnerable child, are persistent, distressing to others or are resistant to normal parental correction and boundary setting. |
| Bromberg & O'Donohue | 2013 | UK | Book | Children and adolescents | Book on sexuality in children and adolescents, including chapters on problem sexual behaviour and sexual offending. | Sexual behaviour problems are child-initiated behaviours related to physical boundaries or sexual body parts that are developmentally inappropriate or potentially physically or emotionally harmful to the child or others (Silovsky & Bonner, 2003). |
| Clionsky & N'Zi | 2019 | USA | Review | Adolescents with autism spectrum disorder who engage in HSB. | Developmental and sexual assessment is crucial to assessment and treatment. | Children (8-12) who initiate a specific set of behaviours involving sexual body parts that is developmentally inappropriate and/or potentially harmful, between peers and/or adults and related to curiosity, anxiety, imitation, attention seeking, self-soothing, or other reasons (Silovsky & Bonner, 2003). |
| DeLago et al. | 2020 | USA | Cross-sectional | Children who had experienced inappropriate sexual contact by another child | Most of the children who had initiated the inappropriate sexual contact were male, related to the victim, and had experienced multiple adverse events. | Children diagnosed with having experienced inappropriate sexual contact initiated by another child when the other engaged in age-inappropriate sexual behaviours using coercion or control, or if the other child was developmentally dissimilar, defined by differences in cognitive ability, or an age difference of 4 or more years (Kellogg & the Committee on Child Abuse and Neglect, 2009). |
| Elkovitch et al. | 2009 | USA | Review | Children with sexual behaviour problems. | Provides an overview of development and risks of sexual behaviour problems in prepubescent children. | Children ages 12 and younger who initiate behaviours involving sexual body parts that are developmentally inappropriate or potentially harmful to themselves or others (Chaffin et al., 2008). |
| El-Murr | 2017 | Australia | Review | Children and young people with problem and abusive sexual behaviours. | Review of the literature on problem and abusive sexual behaviours in Australian children and young people. | Problem sexual behaviours applies to children under 10 years old, who are below the age of criminal responsibility. Describes sexual behaviours that are outside the typical range for age and/or stage of development (CEASE, 2016). |
| Fonagy et al. | 2015 | UK | Randomised control trial | 8 families from London. | Study designed to assess the effectiveness of MST-PSB compared to management as usual. Outcomes not reported in this paper. | Problematic sexual behaviour in adolescents is sexual behaviour that is developmentally inappropriate or potentially harmful to self or others and includes behaviour of a sexual nature that infringes on the rights of others (such as the use of coercion or force; Tarren-Sweeney, 2008). |
| Gray et al. | 1997 | USA | Cross-sectional | 6–12-year-olds who had engaged in PSB and their families. | High rates of parental and familial distress including violence, sexual victimisation and perpetration, physical abuse, parental arrest, poverty, denial, disability, and prior therapy were present in this group. | Sexual behaviours defined as problematic because they were (1) repetitive, (2) unresponsive to adult intervention and supervision, (3) equivalent to a criminal violation if performed by an adult, (4) pervasive, occurring across time and situations, (5) diverse array of sexual acts. |
| Grossi et al. | 2016 | USA | Retrospective archival study | Children in a high-risk child welfare sample. | Inconsistent definitions and interpretations of sexual behaviours in children made it difficult to study. | Sexual behaviours outside of socially acceptable boundaries that are exhibited for a number of reasons, including sexual gratification, self-soothing, and attention seeking (Chaffin et al, 2008). |
| Hall et al. | 2000 | Canada | Cross-sectional | 3-7 year old children who had been sexually abused. | 5 profiles of sexual behaviour emerged. These groups differed in most areas of child and family functioning, including treatment outcomes. | 4 groups based on 'appropriateness' of sexual behaviours and key factors for the instigating child (Johnson & Feldmeth, 1993).  5 profiles identified in analysis; 1. Developmentally expected and developmentally problematic, 2. Interpersonal, unplanned, 3. Self-focussed, 4. Interpersonal, planned (noncoercive), 5. Interpersonal, planned (coercive). |
| Hawkes | 2010 | UK | Case study | Boys under 10 referred to specialist service. | Prior neglect and non-sexual forms of maltreatment were common in children causing harm, all had experienced child sexual abuse. | Typology (a) reactive, (b) coercive, punitive, or compulsive attachment and (c) calculated, sexually aroused |
| Horton | 1996 | USA | Review | Children with sexual behaviour problems. | Several factors were identified that contribute to the development of sexual behaviour problems in children. | Sexual behaviours which are disturbing to others or have negative consequences for the child such as public masturbation, exposure of genitals, sexual talk, precocious sexual activity with other children, and coercive sexual behaviour with others. |
| Jenkins et al. | 2020 | USA | Prettest-posttest | Young people with PSB. | Low intensity outpatient treatment may be effective for this group. | Youth-initiated behaviours which are developmentally inappropriate and/or potentially harmful (Silovsky, 2012). |
| Johnson | 2002 | USA | Literature review | Children and young people with different types of sexual behaviours | Distinguishing between groups is important for informing appropriate responses. | 4 groups along a continuum, (1) healthy sexual behaviour, (2), sexually-reactive children, (3) children who engage in extensive, mutual sexual behaviours, (4) children who molest other children (Johnson, 1988, 1999, 2000). |
| Kulesz & Wyse | 2007 | USA | Cross-sectional | Children who had been sexually abused. | Early identification and treatment may prevent the victimisation of other children. | Problematic sexual behaviours are those exhibited by children that are not normal sexual exploration. May be categorised into 3 groups, sexually reactive behaviours, extensive mutual sexual behaviours, or children who molest other children (Johnson, 1993). |
| Louis et al. | 2016 | USA | Book | Children with sexual behaviour problems. | Reviews play therapy strategies for children, including those with sexual behaviour problems. | Children 12 years old or younger who demonstrate developmentally inappropriate or aggressive sexual behaviour (National Centre on Sexual Behaviour of Youth, 2003). |
| Lévesque et al. | 2012 | Canada | Cross-sectional | Children with sexual behaviour problems. | When PSB and gender were controlled, verbal abuse and neglect emerged as predictors of externalising problems. | Behaviours become problematic when they begin to involve body parts (e.g. genitals, anus, buttocks, and breasts) and to be developmentally inappropriate or potentially harmful to them or others (ATSA, 2006). |
| Lévesque et al. | 2010 | Canada | Cross-sectional | Children with sexual behaviour problems | When age was controlled, greater exposure to sexualised behaviours in the family was both a correlate and a predictor of PSB persistence in children. Externalising problems and somatic complaints were also correlates of PSB. | Behaviours become problematic when they begin to involve body parts (e.g. genitals, anus, buttocks, and breasts) and to be developmentally inappropriate or potentially harmful to them or others (ATSA, 2006). |
| Mangold et al. | 2021 | USA | Cross-sectional | Adults. | Clinician's would likely benefit from multiple informants and considering adult's prior experiences with CSA when evaluating reports of PSB. | May pose a risk to the safety and well-being of the initiating children and the other children involved. Sexual behaviours are considered problematic when they involve threats, force, or aggression; children of disparate ages; or provoke strong emotional reactions in either child (American Academy of Pediatrics, 2005). |
| McInnes & Ey | 2020 | Australia | Exploratory project | Educators and carers of primary school aged children. | Educators and carers reported a low level of understanding about PSB in primary school aged children and concern regarding current responses. | Behaviours of a sexual nature displayed by or between children which are age inappropriate, harmful, and occurring between children who are younger that the age of criminal culpability. Behaviours typically include elements of aggression, secrecy, coercion and are outside the range of what is defined as age-appropriate sexual behaviour, and often feature a substantial difference in age or development between participants (Hackett, 2011). |
| Mesman et al. | 2019 | USA | Review and case study | Health care professionals. | Frequency of the behaviour, developmental considerations, and the level of harm may help health care professionals assess whether the sexual behaviour is typical or problematic. | Actions that children aged 12 years and younger engage in that are developmentally inappropriate, involve sexual body parts, and may be harmful to themselves or others (Chaffin et al., 2008). |
| Morrill & Bachman | 2013 | USA | Exploratory | Undergraduate and graduate students. | No gender differences relating to surviving sibling abuse or perpetrating emotional and physical abuse, whereas women had a significantly higher rate of perpetration of sibling sexual abuse. | Sexual behaviour between siblings that is not age appropriate, not transitory, and not motivated by developmentally appropriate curiosity (Caffaro & Con-Caffaro, 1998). |
| National Centre on Child, Abuse and Neglect | 1991 | USA | Report. | Families. | Report on family violence issues. | Sexual acts between siblings, when one sibling is significantly older than the other, are considered sexual abuse. |
| O'Brien | 1991 | USA | Book | Siblings. | Book chapter on sibling incest. | Sexual behaviour between siblings that is exploitative in nature due to a significant age difference or where force, violence, or intimidation is employed. |
| Offermann et al. | 2008 | USA | Cross-sectional | Children who have been sexually abused and now display PSB. | Preliminary results support effectiveness of SMART model in targeting PSB symptoms in young, sexually abused children. | Sexual behaviour is problematic if it placed the child at risk or violated social norms, behaviours that interfere with the child's development and relationships cause others to feel uncomfortable or are abusive or coercive indicate a need for therapeutic intervention (Ryan, 2000a, 2000b). |
| Pourliakas et al. | 2016 | Australia | Rapid evidence review. | Children and young people in out-of-home care. | CBT may have some benefit, but MST may have greater gains in the treatment of children engaged in PSB. | Sexual behaviour that is outside the normal developmental activity and has a detrimental effect on a child or young person's engagement in everyday tasks (NSW Department of Health, 2005; Staiger, 2005). |
| Pithers et al. | 1998 | Australia | Cross-sectional | Children with PSB. | Five distinct groups emerged from analysis that may help inform treatment - sexually aggressive, non-symptomatic, highly traumatised, rule breaker, abuse reactive. | 3 types (a) inappropriate sexual behaviour (theoretically might not suggest psychological disturbance or the need for intensive intervention, e.g. public masturbation, exposure, sexualised play), (b) developmentally precocious behaviour (developmentally unexpected attempts at intercourse that did not entail use of force), and (c) coercive sexual behaviour (involves use of force, or implied force, to gain victim submission (Berliner et al., 1986). |
| Pitre et al. | 2021 | France | Cross-sectional | Children with PSB. | Heterogenous population, many have experienced significant challenges (e.g. victimisation, placements, other behaviour problems). Treatment resulted in significant decreases in the most problematic sexual behaviours, as well as other internalised and externalised behaviour problems. | Children aged 12 and younger who initiate behaviours involving sexual body parts (i.e. genitals, anus, buttocks, breasts) that are developmentally inappropriate or potentially harmful to themselves or others (ATSA, 2006). |
| Pritchard et al. | 2011 | UK | Case study. | 1 x 16 year old boy and associated services. | Significant results not found until week 49 when multiple component intervention used. Long term treatment may be necessary for those with learning disabilities. Restricting community visits alone was not affective. | Accessing pornography, sexual comments, inappropriate touching, following boys and girls. |
| Rasmussen | 1999 | USA | Review | Children with sexually abusive behaviour problems. | Trauma Outcome Process addresses treatment issues of both victims and offenders and adds useful insight in understanding these issues by describing self-destructive, abusive, and adaptive responses to traumatic experiences. | Sexual behaviours problems create risk for themselves, make others uncomfortable, interfere with psychosocial development, but fall short of being abusive (National Task Force on Juvenile Sex Offending, 1993). |
| Shawler et al. | 2018 | USA | Conceptual overview | Children with PSB. | Parent training is a core component of effective treatments for PSB. | PSB is characterised as developmentally inappropriate or potentially harmful behaviour that involves the use of sexual body parts (Chaffin et al., 2008). |
| Shawler et al. | 2020 | USA | Review | Community members | Victim disclosure and witnessing PSB were the most common identification pathways. Caregivers and school personnel were the most common initial identifiers. | PSB of youth is defined as sexual behaviours that occur across childhood that fall outside what is developmentally expected, involve a sexual body part, and may cause harm to the youth or others (Chaffin et al., 2008). |
| Silovsky et al. | 2020 | USA | Introduction paper, special issue, on children with PSB | Children with PSB | Provides an overview for a special issue on children with PSB. | Sexual behaviour of a child that is developmentally inappropriate and potentially harmful to themselves or others (Silovsky & Bonner, 2003). |
| Silovsky & Niec | 2002 | USA | Case review | Young children with sexual behaviour problems | Significant emotional and behavioural problems were evident, and caregivers reported high levels of stress relating to parenting. Most of the children were female (65%), contrary to findings in school-aged samples. | Sexual behaviour in children is defined as a problem when it (a) occurs at a greater frequency or at a much earlier age than would be developmentally expected, (b) interferes with children's development, (c) occurs with the use of coercion, intimidation or force, (d) is associated with emotional distress, and/or (e) reoccurs in secrecy after intervention by caregivers (Hall et al., 1996). |
| Silovsky et al. | 2013 | USA | Book | Children and youth with sexual behaviour problems | Book chapters on sexual behaviour problems in children. | Child initiated behaviours related to physical boundaries or sexual body parts that are developmentally inappropriate or potentially physically or emotionally harmful to the child or others (Silovsky & Bonner, 2003). |
| Silovsky et al. | 2019 | USA | Quasi-experimental | Youth with PSB | Early intervention important in improving outcomes for families and reducing risk for further harm. | Behaviour involving sexual body parts that are developmentally inappropriate or potentially harmful to themselves or others (Swisher et al., 2008). |
| Slemaker et al. | 2021 | USA | Qualitative | Child victims of youths with PSB | One of the main themes identified was barriers to identifying victims and their subsequent access to mental health services. | Sexual behaviours that are developmentally inappropriate and potentially harmful to the child themselves or others (Chaffin et al., 2008). |
| Smith et al. | 2019 | USA | Case review | Pre-school aged children with intrusive sexual behaviours. | ISB was most strongly related to variables of the child's vulnerability such as higher levels of general externalising behaviours and PTSD symptom severity. | PSBs are clinically concerning behaviours that involve sexual body parts that are developmentally inappropriate and/or potentially harmful (Silovsky & Bonner, 2003).  ISB defined as a subtype of PSB that are categorised as invasive and/or aggressive (e.g. touching others private parts, attempting intercourse, use of force; Friedrich, 1997, 2002). |
| Szanto et al. | 2012 | USA | Case review | Children and adolescents with PSB in state custody. | Sexual abuse and multiple trauma experiences appear to have an important possible etiological role in the developmental of PSB. | Children ages 12 and younger who initiate behaviours involving sexual body parts that are developmentally inappropriate or potentially harmful to themselves or others (Chaffin et al., 2008). |
| Webster | 2019 | Australia | Case studies | Children and young people with PSB or HSB. | Analyses two case studies of prepubescent boys with PSB. Examining unconscious and conscious procedures, motives, and meaning of children expands professional understanding. | 3 circumstances in which sexual behaviour can be problematic: (a) where a child is psychologically harmed by the behaviour; (b) where the child’s behaviour places him or her outside societal norms, and (c) where the behaviour is abusive (Staiger, 2005). Sexual behaviour is problematic when it causes others to feel uncomfortable, occurs at the wrong time or place, and/or in conflicts with family or community values. |
| Webster & Butcher | 2012 | USA and Australia | Review | Children with PSB. | Concludes that a human rights centred approach calls for professionals to recognise, respond to, and reflect upon children’s sexual behaviours in a way that prioritises the dignity of the child. | Johnson’s (1991) 4 clusters of groups of children under 13; 1. Children engaging in natural and healthy childhood sexual play, 2. Sexually-reactive behaviours, 3. Extensive mutual sexual behaviours, 4. Molestation behaviour. Ryan & Lane’s (1997) model of categorising behaviours according to severity – green flag, yellow flag, red flag, and abusive behaviours. |
| Wiche | 1997 | USA | Book | Sibling sexual abuse. | Provides an overview of a range of abusive behaviours between siblings. | Inappropriate sexual contact such as unwanted touching, fondling, indecent exposure, attempted penetration, intercourse, rape, or sodomy between siblings. |

**Table 4. Included Papers that Used Terms Related to HSB**

| **Author(s)** | **Year** | **Region** | **Paper Type/Study Design** | **Population** | **Key Findings** | **Definition** |
| --- | --- | --- | --- | --- | --- | --- |
| Alemango et al. | 2009 | USA | Cross-sectional | Adolescents who had been arrested. | Measured associated risk factors such as drug use, mental and physical health, physical violence and found that drug use was important to consider in treatment particularly for females. | Risky sexual behaviour defined as a first sexual experience younger than 13, unprotected sex, sex while drunk or high, or exchanging sex for anything. |
| Alexy et al. | 2009 | USA | Cross-sectional | Sexually reactive children and young people. | SRCA who used pornography were more likely to display aggressive behaviours than those who did not access pornography. | SRCAs are individuals who engage in sexually inappropriate and/or coercive acts with other youth and adults (Araji, 1997; Prentky et al., 2003; Ray et al., 1995). |
| Anderson & Parkinson | 2018 | Belgium & UK | Review | Children and young people engaged in HSB. | Review of the research and practice for family group conferences relating to HSB. | HSB encompasses a wide range of sexually abusive and harmful acts. These contact and non-contact behaviours range from rape and extreme sexual violence to indecent exposure and voyeurism (Grimshaw,2008). HSB is distinct because it is perpetrated by a young person (under 18 years old) and such behaviours are always problematic. |
| Armstrong | 2021 | Scotland | Review | Children and young people engaged in HSB. | Review of the criminal justice system in Scotland and other international restorative programmes. There are difficulties present in balancing the rights of the victim and the child or young person who perpetrated the HSB. | Sexual behaviours expressed by children and young people under the age of 18 years old that are developmentally inappropriate, may be harmful towards self or others, or be abusive towards another child, young person, or adult (Hackett et al., 2016). |
| Australian Government | 2017 | Australia | Royal Commission Final Report | Australian adult survivors of child abuse in institutional settings | Understand the nature, prevalence, and impact of child sexual abuse in Australian institutions. | HSB as an umbrella term to describe a range of sexual behaviours in children and young people including PSB and sexual offending. PSB defined as sexual behaviours outside the normal or age-appropriate range for younger children and may or may not result in harm. Sexual offending defined as sexual behaviour in those over the age of 10 that is a sexual offence. |
| Banks | 2014 | UK | Review | Adolescents engaged in HSB | More training is needed for schools to manage HSB between peers. Special schools in particular had regular incidents of peer sexually abusive behaviours. | Non-consenting sexual acts which resulted in trauma on the part of the victim (Fyson, 2005). |
| Barry & Harris | 2019 | UK | Cross-sectional | Children and their parents/carers engaging in group and family interventions for PSB or HSB | The CBT group intervention was largely beneficial in reducing PSB and HSB. | Sexual behaviour can be problematic (not appropriate for the child's age or development), as well as harmful, particularly if the behaviour involves coercion and power over a victim (Hackett, 2014). |
| Cranbourne-Rosser et al. | 2020 | UK | Review | Children and young people who had engaged in HSB. | Presence enhances therapeutic relationship and connectivity. | Sexual behaviours expressed by children and young people under the age of 18 years old that are developmentally inappropriate, may be harmful towards self and others, or be abusive towards another child, young person or adult (Hackett et al., 2016). |
| De Crisce | 2013 | USA | Review | Adolescents. | A review of sexual addiction and hypersexual behaviours in adolescents. | 4 categories - normal adolescent sexual behaviour, behaviour requiring adult response, behaviour requiring correction and illegal behaviours requiring immediate intervention (Ryan, 2000a). |
| Draugedalen | 2021 | Norway | Cross-sectional | Teachers in primary schools in Norway. | An overwhelming majority of teachers reported that they had not received any knowledge, information, or training related to HSB. | Sexual behaviours of children and young people under the age of 18 years old that are developmentally inappropriate, may be harmful toward self or others, or be abusive towards another child, young person or adult (Hackett, 2014). |
| Draugedalen et al. | 2021 | Norway | Qualitative | Teachers in primary schools in Norway. | Primary school teachers reported a lack of HSB competency and limited multi-agency cooperation. | Sexual behaviours of children and young people under the age of 18 years old that are developmentally inappropriate, may be harmful toward self or others, or be abusive towards another child, young person or adult (Hackett, 2014). |
| El-Murr | 2017 | Australia | Review | Children and young people with problem and abusive sexual behaviours. | Review of the literature on problem and abusive sexual behaviours in Australian children and young people. | Sexually abusive behaviour applies to children and young people aged 10-17, defined by criminal law to be minors but who have reached the minimum age of criminal responsibility (CEASE, 2016). |
| Firmin | 2020 | UK | Qualitative | UK staff and students. | Both staff and students were found to reinforce norms of HSB and in doing so created contexts conducive with peer-sexual abuse. | Hackett’s (2011) continuum of HSB. |
| Gibson | 2014 | UK | Review | Young people engaged in HSB. | Involving young people in safety planning may be a way to improve this practice. | Any form of sexual activity with another individual, that they have powers over by virtue of age, emotional maturity, gender, physical strength, intellect, and where the victim in this relationship has suffered sexual exploitation and betrayal of trust (Calder et al., 2001). |
| Grossi et al. | 2017 | USA | Retrospective longitudinal archival design | Male children and adolescents involved in child welfare system in Massachusetts. | Youths who exhibited PSBs first in early childhood had higher sexual re-offense rates than youths who exhibited these behaviours in middle childhood or preadolescence/adolescence. | Sexually inappropriate and/or coercive behaviours, ranging from exposure of genitalia to forcible rape (Chaffin et al., 2008). |
| Hackett | 2014 | UK | Report | Children and young people with HSB. | Provides an overview of the literature on children and young people with HSB. | Sexual behaviour expressed by children and young people under the age of 18 years old that are developmentally inappropriate, may be harmful towards self or others, or be abusive towards another child, young person, or adult (Hackett, 2014). |
| Hallett et al. | 2019 | Wales | Cross-sectional | Young people with HSB and those who had experienced child sexual exploitation (CSE). | Gendered differences between those engaging in HSB and those who have experienced CSE. Experiences of abuse, family history of domestic violence were nearly identical between groups. | Sexual behaviours expressed by children and young people under the age of 18 years old that are developmentally inappropriate, may be harmful towards self or others, and/or be abusive towards another child, young person or adult (Hackett, 2014). |
| Hatton | 2019 | UK | Case study | Young people with HSB. | Adapting sessions to meet client needs is important which could include addressing minimisation, perspective taking, and HSB consequences. | Contact HSB involves forcing or enticing another person to engage in penetrative or non-penetrative sexual activity. Non-contact HSB does not involve touch such as distributing indecent photographs of children or indecent exposure (Sanderson, 2006). |
| Hunter | 2011 | USA | Book | Young people with HSB. | Book designed for therapists working with young people with sexual behaviour problems. | Youths who engage in non-consensual sexual behaviour (consent defined as either having sex with a significantly younger youth or that which is coercive or manipulative in nature), both are harmful to the victim. |
| Jensen et al. | 2020 | Norway | Cross-sectional | Younger child and peer victims of HSB. | Adolescents who offended against younger children were more likely to have experienced trauma earlier in life, have multiple victims, and repeated behaviours compared to those who had offended against peers. | One or more children engaging in sexual discussions or acts that are inappropriate for their age or stage of development. This can range from using sexually explicit words and phrases, inappropriate touching, using sexual violence or threats, to full penetrative sex with other children and adults (NICE, 2016). |
| King-Hill | 2021 | UK | Mixed methods | Young people with HSB. | The Traffic Light tool was found to enhance practitioner’s confidence, but can remove consideration of context. | Sexual behaviours expressed by children and young people under the age of 18 years old that are developmentally inappropriate, may be harmful towards self and others, or be abusive towards another child, young person or adult (Hackett et al., 2019). |
| Lewis | 2018 | UK | Review | Young people engaged in technology assisted HSB. | Atypical sexual interest may be most important target for intervention. Many young people had first been exposed to these behaviours by an adult. | Behaviours that are likely to cause distress to others due to significant differences in age, development or power, or where the behaviours are excessive, forceful, planned and/or secretive (Brook, 2012). |
| Lloyd | 2019 | UK | Mixed methods | Young people with HSB. | Responses and interventions for HSB in schools need to move beyond individual behaviours to intervening within factors within the school themselves. | Sexual behaviour expressed by children and young people under the age of 18 years old that are developmentally inappropriate, may be harmful towards self or others, or be abusive towards another child, young person, or adult (Hackett, 2014). |
| Lloyd et al. | 2020 | UK | Mixed methods | Young people with HSB. | Assessing the nature of HSB between students in relation to context (e.g. schools) is important in informing appropriate responses. | Sexual behaviour expressed by children and young people under the age of 18 years old that are developmentally inappropriate, may be harmful towards self or others, or be abusive towards another child, young person, or adult (Hackett, 2014). |
| Malovic et al. | 2020 | UK | Systematic review | Young with HSB and intellectual disabilities. | Those with intellectual disabilities tend to more opportunistic and less complex in their offending behaviours. | Behaviours (verbal or physical) between two or more persons which are inappropriate given the ages and/or developmental stages of the participants (Rich, 2011). |
| Malvaso et al. | 2020 | Australia | Systematic literature review | Children and young people displaying sexually abusive behaviour. | ASOs access victims through 4 main channels, routine daily activities, personal relationships, an organisational role, and the internet. Further research is needed for internet HSB. | Sexually abusive behaviour describes behaviours of young people aged 10-17 that occurs in the context of the absence of consent, and which may involve the use of threat of force, or force and coercion (CEASE, 2012). |
| Marriage et al. | 2017 | Australia | Cross-sectional | Parents. | Parents were significantly less able to identify and respond to behaviours exhibited by very young children (0-4 age bracket). When parents viewed both the victim and perpetrator as being their children (siblings) they reported lower intervention response levels | Green sexual behaviours are "normal, age appropriate, spontaneous, curious, mutual, light-hearted, and easily diverted experimentation", orange are "outside normal behaviour in terms of persistence, frequency or inequality of age, power or ability, and signal the need to monitor and provide extra support", red sexual behaviours are "problematic or harmful, forceful, secretive, compulsive, coercive or degrading, and signal the need to provide immediate protection and follow-up support" (Family Planning Queensland, 2012). |
| McKibbin | 2017 | Australia | Scoping review | Young people with HSB living in residential care. | The current response to prevention is under-developed. Educative interventions, targeting grooming and PSB, and providing a holistic response was discussed. | Sexual behaviour carried out by children and young people that is developmentally inappropriate and abusive towards self or others (Hackett et al., 2016). |
| McKibbin & Humphreys | 2021 | Australia | Scoping review | Frontline workers. | A public health approach would enable linking between prevention and response strategies. | Behaviour undertaken by children and young people that is developmentally inappropriate and may involve the use of coercion or abuse (Hackett et al., 2016). |
| McKibbin et al. | 2022 | Australia | Review | Children and young people in out-of-home care. | A gap exists in secondary prevention efforts for children and young people living in residential care. | Sexual behaviour carried out by children and young people that is developmentally inappropriate and abusive towards self or others (Hackett et al., 2016). |
| McKibbin & Humphreys | 2019 | Australia | Qualitative | Children and young people in out-of-home care | Prevention and response strategies must take into account challenges for children and young people in out of home care. | Child sexual exploitation is a form of child abuse that occurs when an individual or group takes advantage of an imbalance of power to coerce, manipulate or deceive a child or young person under the age of 18 into sexual activity (a) in exchange for something the victim needs or wants and/or (b) for the financial advantage or increased status of the perpetrator or facilitator. The victim may have been sexually exploited even if the sexual activity appears consensual and does not always involve physical contact, as it can also occur through the use of technology (Beckett et al., 2017). |
| McKibbin et al | 2017 | Australia | Qualitative | Young people with HSB | Three opportunities for prevention identified, reform sexuality education, redress their victimisation experiences, and help their management of pornography. | The involvement of a child in sexual activity that he or she does not fully comprehend, is unable to give informed consent to, or for which the child is not developmentally prepared, or else that violates the laws or social taboos of society. Children can be sexually abused by both adults and other children who are—by virtue of their age or stage of development—in a position of responsibility, trust or power over the victim (World Health Organisations, 2006). |
| Meiksans & Bromfield | 2017 | Australia | Report, literature review | Children and young people with HSB | Government report reviewing HSB research. | Any behaviour of a sexual nature expressed by children under 18 years old that is outside of what is culturally accepted as typical sexual development and expression, is obsessive, coercive, aggressive, degrading, violent, or causes harm to the child or others, involves a substantial difference in age or developmental ability of participants (Evertsz & Miller, 2012; Hackett et al., 2016). |
| Pelech et al. | 2021 | UK | Systematic review | Children and young people with HSB. | Professionals working with children and young people engaged in HSB report a range of feelings associated with their work. Clinical supervision was identified as important. | Behaviour involving sexual body parts that are developmentally inappropriate or potentially harmful to themselves or others (Chaffin et al., 2008). |
| Rasmussen | 1999 | USA | Review | Children with sexually abusive behaviour problems | Trauma Outcome Process addresses treatment issues of both victims and offenders and adds useful insight in understanding these issues by describing self-destructive, abusive, and adaptive responses to traumatic experiences. | Coercive sexual behaviours far beyond developmentally appropriate childhood exploration (Friedrich, 1993). |
| Russell & Harvey | 2016 | UK | Cross-sectional qualitative. | Staff members from a Sexual Behaviour Service. | Staff appreciated factors contributing to sexually harmful behaviour and experienced challenges. Clinical supervision identified as important to support staff. | Sexually harmful behaviour displayed by adolescents may involve age-inappropriate behaviour such as the use of explicit language, inappropriate touching, or violence with a sexual nature. |
| Ryan et al. | 2012 | USA | Book | Juvenile sex offenders. | Book chapter on juvenile sex offenders. | Sexually abusive behaviour as any sexual behaviour that occurs without consent, without equality, or as a result of coercion (National Task Force on Juvenile Sex Offending, 1993). |
| Shaw & Antia | 2010 | USA | Book | Sexually aggressive youth. | Book chapter on sexually aggressive youth. | Sexually abusive behaviour occurs without consent, without equality, or as a result of coercion (National Task Force on Juvenile Sex Offending, 1993). |
| Taylor et al. | 2020 | USA | Qualitative | Professionals. | Professionals perceived youth's engagement in electronic and online sexual behaviours to be frequent, growing, and concerning yet reported that they lacked clear protocols to determine severity of the behaviours and guide responses. | Behaviours that involve sexual body parts in a manner that is developmentally inappropriate or potentially harmful to the youth or others (Chaffin et al., 2008). |
| Taylor et al. | 2021 | USA | Qualitative | Professionals working in Children's Advocacy Centres. | All sites perceived sibling HSB as a family crisis, prioritising safety and therapeutic interventions. Parents were identified as key factors in addressing safety. | Sexual behaviour exhibited by children and adolescents that is developmentally inappropriate and/or harmful or abusive towards their self or others (Hackett et al., 2016). |
| Tener et al. | 2022 | Israel | Qualitative | Adult survivors of HPSB. | Both the HPSB experiences themselves and the subsequent disclosure contributed to the development of detached, mistrustful identities among the participants. | Childhood sexual abuse and assault or peer victimisation (Finkelhor et al., 2013).  PSB refer to children engaging in actions that are developmentally inappropriate, involve sexual body parts, and may be harmful to themselves or others (Chaffin et al., 2008). |
| van Eys | 1997 | USA | Review | Pre-pubescent boys with sexually aggressive behaviour. | Provides a summary of key findings of treatment approaches for pre-pubescent boys with sexually aggressive behaviour. | 4 groups based on 'appropriateness' of sexual behaviours and key factors for the instigating child (Johnson & Feldmeth, 1993). |
| Waters et al. | 2021 | UK | Review | Teachers. | Teachers identified a lack of knowledge, training, and support in relation to HSB. | Aggressive or manipulative sexual interactions that occur without the consent of the victim (NSPCC, 2002). |
| Yates | 2018 | UK | Qualitative, constructivist grounded theory | Social workers. | Social workers were found to make decisions intuitively, influenced by the mindset that siblings are better together. A risk to this approach is victim children becoming lost in responses and interventions. | Children engaging in any form of sexual activity with another individual that they have powers over by virtue of age, emotional maturity, gender, physical strength, intellect, and where the victim in this relationship has suffered a sexual exploitation (Calder, 1999). |
